# Iron overload adversely effects bone marrow haematogenesis via SIRT-SOD2-mROS in a process ameliorated by curcumin

**DOI:** 10.1186/s11658-020-00244-7

**Published:** 2021-01-13

**Authors:** Shujuan Zhou, Lan Sun, Shanhu Qian, Yongyong Ma, Ruye Ma, Yuqing Dong, Yifen Shi, Songfu Jiang, Haige Ye, Zhijian Shen, Shenghui Zhang, Jianping Shen, Kang Yu, Siqian Wang

**Affiliations:** 1 Department of Haematology, The First Affiliated Hospital of Wenzhou Medical University, Wenzhou, 325000 Zhejiang People’s Republic of China; 2 Department of Haematology, The First Affiliated Hospital of Zhejiang Chinese Medical University; The First Clinical Medical College of Zhejiang Chinese Medical University, Hangzhou, 310006 Zhejiang People’s Republic of China; 3 Department of Prosthodontics, School & Hospital of Stomatology, Wenzhou Medical University, Wenzhou, 325000 Zhejiang People’s Republic of China

**Keywords:** Curcumin, Iron overload, Bone marrow damage, Autophagy, Mitochondrial ROS

## Abstract

**Background:**

Iron overload, which is common in patients with haematological disorders, is known to have a suppressive effect on haematogenesis. However, the mechanism for this effect is still unclear. The antioxidant curcumin has been reported to protect against iron overload-induced bone marrow damage through an as-yet-unknown mechanism.

**Methods:**

We established iron overload cell and mouse models. Mitochondrial reactive oxygen species (mROS) levels, autophagy levels and the SIRT3/SOD2 pathway were examined in the models and in the bone marrow of patients with iron overload.

**Results:**

Iron overload was shown to depress haematogenesis and induce mitochondrion-derived superoxide anion-dependent autophagic cell death. Iron loading decreased SIRT3 protein expression, promoted an increase in SOD2, and led to the elevation of mROS. Overexpression of SIRT3 reversed these effects. Curcumin treatment ameliorated peripheral blood cells generation, enhanced SIRT3 activity, decreased SOD2 acetylation, inhibited mROS production, and suppressed iron loading-induced autophagy.

**Conclusions:**

Our results suggest that curcumin exerts a protective effect on bone marrow by reducing mROS-stimulated autophagic cell death in a manner dependent on the SIRT3/SOD2 pathway.

## Introduction

Iron overload is common in patients with haematological disorders due to long-term repeated red cell transfusion, congenital iron overload disease, and a subclass of anaemia characterised by ineffective haematogenesis, leading to excess iron deposition throughout the body. This last form causes tissue damage and organ dysfunction and eventually leads to the mortality and morbidity associated with anaemia-related diseases [1, 2]. There is accumulating clinical evidence that iron overload has a suppressive effect on haematogenesis, and that iron chelation therapy can improve this condition [3–7]. The damaging effect of iron overload is due to the elevation in cellular labile iron pools (LIPs), which generate reactive oxygen species (ROS). However, the mechanism by which ROS damage haematopoietic cells remains unclear.

The mitochondria are a focal point of iron metabolism and a major source of ROS [8]. Mitochondrial reactive oxygen species (mROS) have been shown to induce autophagy [9–11]. The molecular mechanism of autophagy has been widely investigated. Sirutin 3 (SIRT3) is the main mitochondrial acetyl-lysine deacetylase. It modulates multiple proteins, thereby controlling mitochondrial function and mROS generation [12]. It directly binds and deacetylates superoxide dismutase 2 (SOD2), which increases that protein’s activity, significantly impacting mROS homeostasis and autophagic flux [13, 14].

Curcumin is a naturally occurring yellow pigment isolated from the rhizomes of *Curcuma longa*. It has been shown to possess anti-inflammatory, antioxidant, and iron-chelating properties [15, 16]. Thus, it shows promise as a therapeutic option in the management of free radical-related diseases [17].

The ability of curcumin to protect against iron overload-induced autophagic cell death in human bone marrow remains unknown. We previously found that iron overload has a deleterious impact on haematogenesis. Here, we examine whether curcumin supplementation can attenuate the haematogenic abnormalities induced by iron overload. We also explore the underlying mechanisms.

## Materials and methods

### Patients and healthy donors

**Fig. 1:**
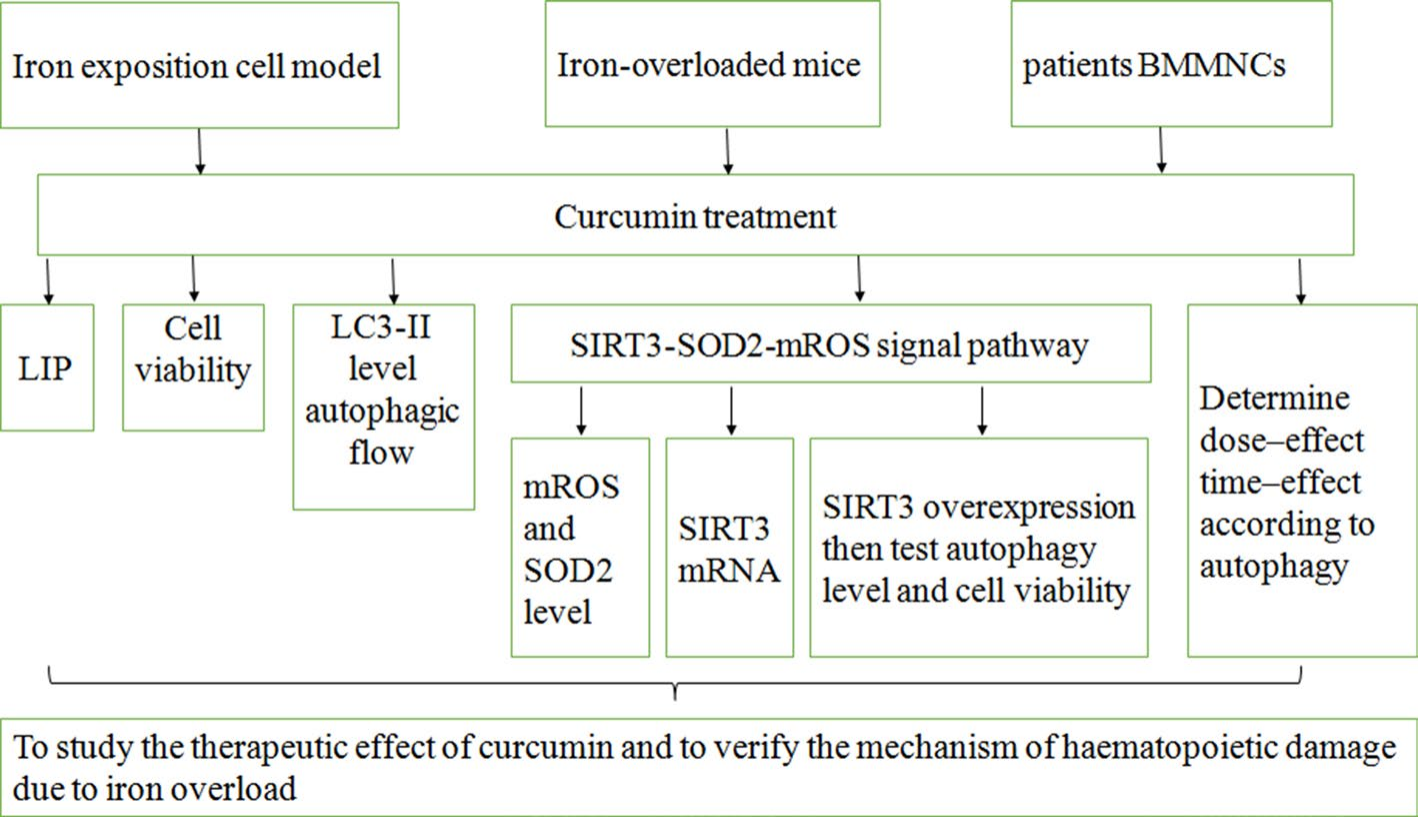
Flowchart of the study. *LIP* labile iron pools, *mROS* mitochondrial reactive oxygen species, *SOD2* superoxide dismutase 2, *SIRT3* sirtuin 3, *BMMNC* bone marrow mononuclear cells

The study population consisted of six patients (three females and three males) diagnosed with thalassemia major and six age-matched healthy subjects. For a detailed flowchart of the study, see Fig. 1. The study design was approved by the Ethics Committee of the First Affiliated Hospital of Wenzhou Medical University. Written consent was obtained from all subjects prior to participation in this study, in accordance with the Declaration of Helsinki.

### Animal studies

Sixty mice weighing 20–25 g were obtained from the Laboratory Animal Centre of Wenzhou Medical University. They had been raised at the Certified Animal Care Facility of the First Affiliated Hospital of Wenzhou Medical University. The mice were randomized into: the iron-overloaded group (200 mg/kg iron dextrin intraperitoneal injection, 1 ml/kg corn oil gavage, n = 15); control group (0.2 ml normal saline intraperitoneal injection, 1 ml/kg corn oil gavage, n = 15); curcumin group (0.2 ml normal saline intraperitoneal injection, 200 mg/kg curcumin dissolved in corn oil gavage, n = 15); and curcumin + iron-overloaded group (200 mg/kg iron dextrin intraperitoneal injection, 200 mg/kg curcumin dissolved in corn oil gavage, n = 15). The iron-overload groups received an intraperitoneal injection of iron dextran every 3 days for 4 weeks. The curcumin groups received a gavage with 200 mg/kg curcumin every day for 4 weeks.

### Peripheral blood cell and BMMNC counts

Using ethylenediaminetetraacetic acid (EDTA) tubes, we collected samples of peripheral blood from the mice via the orbital sinus. Complete blood counts were analysed using a pocH-100i haematology analyser (Sysmex, Kobe, Japan). The cell counts included red blood cells (RBCs), haemoglobin (Hb), white blood cells (WBCs) and platelets (PLTs). The bone marrow mononuclear cells (BMMNCs) were flushed from the bones as described previously by another group [18, 19] and counted using the haematology analyser.

### Cell experiments

BMMNCs were isolated from bone marrow aspirates from healthy donors or patients with thalassemia major using Ficoll-Hypaque density gradient centrifugation. Based on the flow cytometric analyses of CD34 and CD38 expression, the isolates mainly consisted of progenitor and stem cells.

Then, the effects of ferric ammonium citrate (FAC) on autophagy in BMMNCs were evaluated. The cells were treated with FAC (F879; Sigma, USA) at different concentrations (0, 100, 200 and 400 μM) for 24 h.

Next, we investigated the ability of curcumin (targetmol, T516; dissolved in DMSO) to alleviate FAC-induced myelotoxicity. BMMNCs were pre-treated with 30 μM curcumin for 2 h prior to FAC treatment.

Finally, we assessed the role of the SIRT3–SOD2 pathway in mononuclear cell protection after curcumin pre-treatment. Cell viability was analysed using a Cell Counting Kit-8 (CK04; Dojindo Molecular Technologies, Japan) according to the manufacturer’s instructions. Each cell experiment was repeated three times.

### Determination of mROS

To assess mROS levels, BMMNCs were incubated with culture medium containing 10 mM MitoSOX (M36008; Invitrogen, USA) for 20 min at 37 °C. After incubation, fluorescence intensity was measured at an excitation wavelength of 492 nm and an emission wavelength of 595 nm using an Infinite M200 Microplate Reader (Tecan, Switzerland).

### Measurement of SOD2 enzyme activity

SOD2 enzyme activity was assayed using a SOD1 and SOD2 Assay Kit with WST-8 (S0103; Beyotime, China) according to the manufacturer’s instructions. The A_450_ was measured using an Infinite M200 Microplate Reader.

### Plasmids and transfection

The plasmid LV5-SIRT3 was designed by Yuxi Biotechnology (China). Mononuclear cells grown in Dulbecco’s modified Eagle’s medium (DMEM) with 20% foetal bovine serum (FBS) for 24 h were transfected with SIRT3 and control plasmids using Opti-MEM I-reduced serum media and Lipofectamine 2000 according to the manufacturer’s instructions (11668-019; Invitrogen). The cells were washed 24 h after transfection and then processed for immunoblotting and other assays.

### Real-time PCR analysis to detect SIRT3 mRNA

All reagents used for real-time PCR were obtained from Life Technologies (USA). The SIRT3 probes were 5′-GACATTCGGGCTGACGTGAT-3′ and 5′-ACCACATGCAGCAAGAACCTC-3′; and the GAPDH probes were 5′-TGACAACAGCCTCAAGAT-3′ and 5′-GAGTCCTTCCACGATACC-3′.

### SIRT3 activity

SIRT3 enzymatic activity was assayed using a fluorometric kit (BML-AK557-0001; Enzo Life Sciences, USA) according to the manufacturer’s instructions.

### Western blotting analysis

BMMNCs were washed twice and transferred to a new tube and the protein concentrations were determined. The protein samples were separated using SDS-PAGE and transferred onto polyvinylidene difluoride membranes, which were blocked and then incubated overnight at 4 °C with antibodies against microtubule-associated protein 1 light chain 3 (LC3; 1:1000, L7543; Sigma), SIRT3 (1:100, sc-99143; Santa Cruz Biotechnology, USA), SOD2 (1:100, sc-33254; Santa Cruz Biotechnology), and β-actin (1:5,000, A5441; Sigma). The membranes were visualized via enhanced chemiluminescence using Super Signal West Pico blotting detection reagents (34079; Pierce, USA) and exposure to Hyper Performance Chemiluminescence film (Amersham, UK).

### Colony-forming cell assays

To investigate the multipotency of haematopoietic progenitor cells, colony-forming units were assayed using methylcellulose culture medium (Stem Cell Technologies, Canada). Aliquots of 1 × 10^5^ cells were plated in 24-well plates and cultured for 14 days. Colony-forming unit erythroid (CFU-E), burst-forming unit erythroid (BFU-E), colony-forming unit granulocyte–macrophages (CFU-GMs), and colony-forming unit Mix (CFU-Mix) were counted. The cells in each group were seeded in triplicate.

### Intracellular LIP analysis

The cellular LIP level was assessed using calcein-AM fluorescent dye (Sigma) [20]. Briefly, aliquots of 3 × 10^6^ cells were inoculated in 6-well plates. After treatment, the cells were washed twice with PBS and incubated with calcein-AM (CA-AM, 0.125 μmol/l) for 10 min at 37 °C. After washing twice with PBS, the residue was combined with 0.25 μg/ml trypan blue solution and dispersed, and the fluorescence intensity was measured via fluorospectrophotometry with excitation and emission wavelengths of 495 nm and 530 nm, respectively. Next, the samples were incubated with bipyridine (BIP; 100 μM) for 30 min at 37 °C, and the fluorescence intensity was measured again under the same conditions. The difference in cellular fluorescence before and after incubation with BIP reflects the amount of intracellular LIPs.

### Statistical analysis

The results are presented as the means ± SEM and were analysed using *t* test or one-way ANOVA. Data were analysed using GraphPad Prism-5 software (GraphPad Software, USA). In all analyses, p < 0.05 was considered statistically significant.

## Results

### Iron overload can damage bone marrow

In our cell model, we found that iron-overloaded BMMNCs showed decreased cell viability (Additional file 1: Fig. S1A), reduced cell proliferation activity (Additional file 1: Fig. S1B), and significantly increased intracellular LIPs (Additional file 1: Fig. S1C).

In the mouse model, the Hb, PLT count and WBC count were significantly decreased in the peripheral blood (Additional file 2: Fig. S3A). Mononuclear cell activity was lower in the bone marrow of iron-overloaded mice (Additional file 2: Fig. S3B). In addition, there were significantly more LIPs in the mononuclear cells of iron-overloaded mice than in those of the normal control mice (Additional file 2: Fig. S3C).

**Fig. 2:**
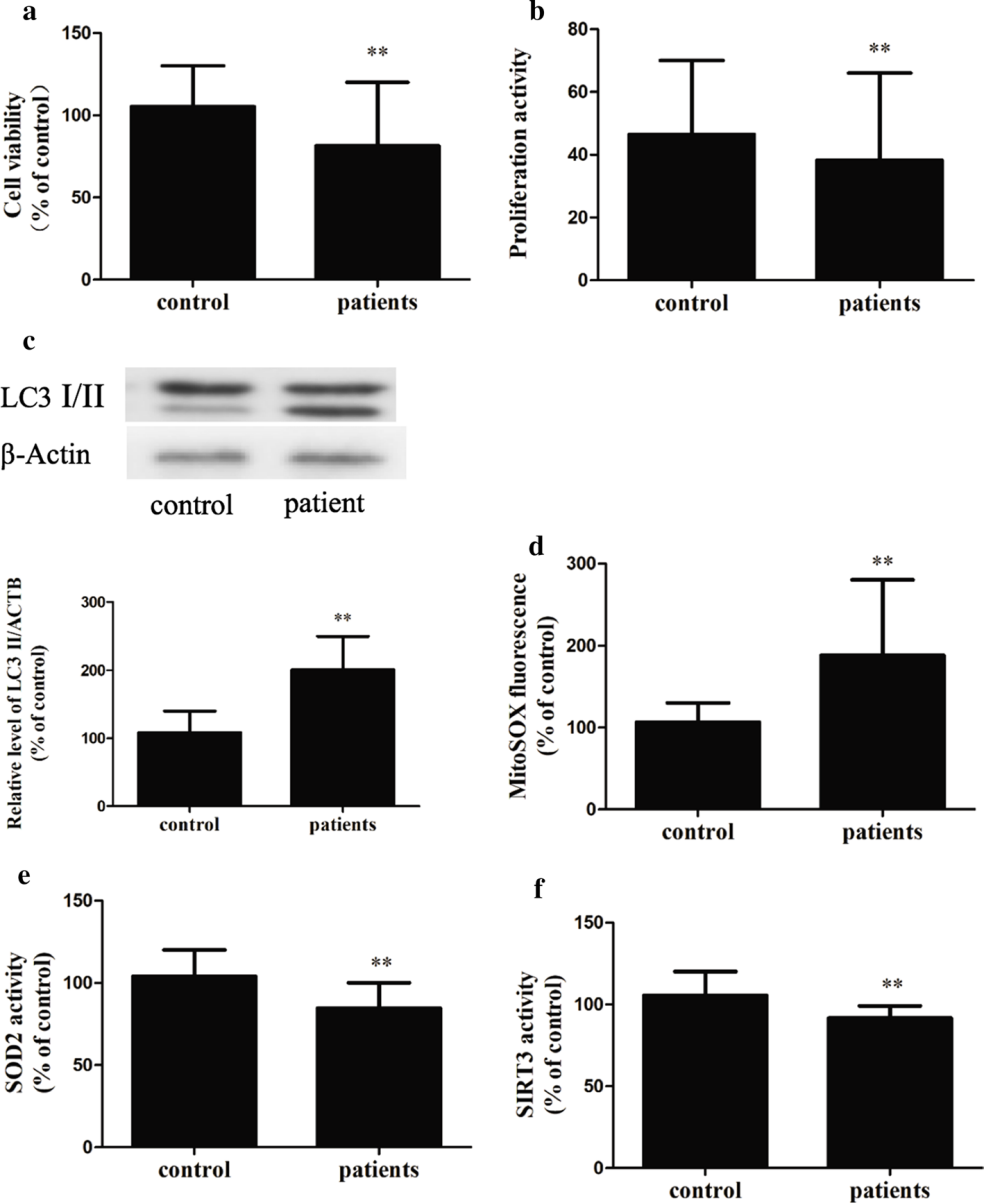
Iron overload (an effect of thalassemia major) affects patients’ bone marrow mononuclear cells. **a**, **b**—The cell viability (**a**) and proliferation activity (**b**) of mononuclear cells in the bone marrow of the patients both decreased. **c** through **f**—LC3 levels (**c**) and mROS levels (**d**) increased, and SOD2 (**e**) and SIRT3 (**f**) activity decreased in the BMMNCs from the patients (n = 6).The results are expressed as a percentage of the control group, which is set at 100%. The values are presented as the means ± SEM, **p < 0.05 vs. the control group

We extracted bone marrow from iron-overloaded patients with thalassemia major and found decreased activity of BMMNCs (Fig. 2a) and reduced proliferative activity of BMMNCs in the bone marrow of these patients (Fig. 2b).

### Iron overload can lead to bone marrow damage through autophagy

**Fig. 3:**
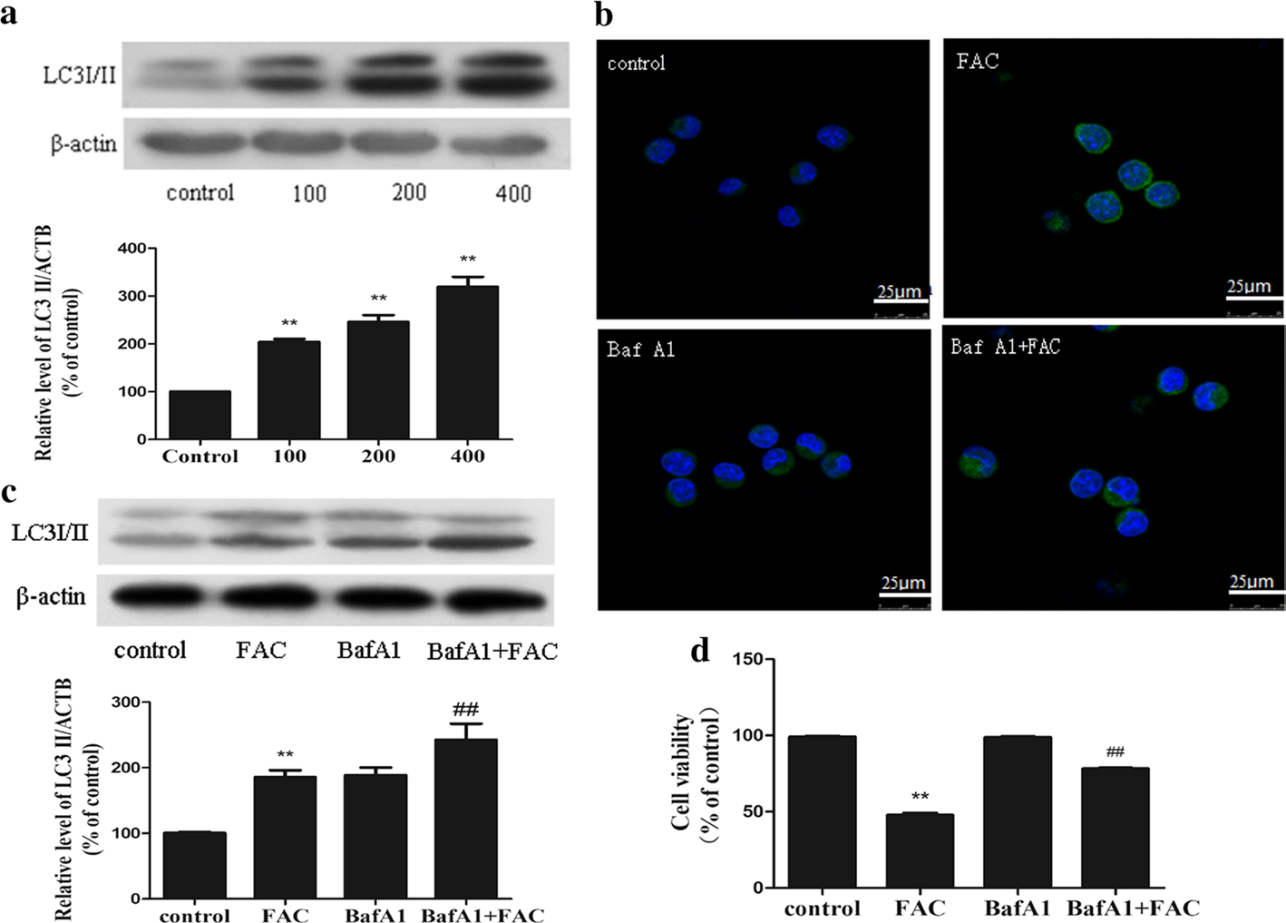
Iron overload can induce autophagic cell death in bone marrow mononuclear cells in vitro. **a**—A representative immunoblot analysis of LC3 assayed after mononuclear cells were treated with FAC at different concentrations (0, 100, 200, 400 μM) for 24 h. β-actin used as an internal standard for protein loading and the experiment was repeated three times. **b**—Confocal microscopy revealed an increased number of autophagic vacuoles when cells were treated with FAC at different concentrations (0, 100, 200, 400 μM) for 24 h. **c**—Bone marrow mononuclear cells were incubated with FAC (200 μM) in the absence or presence of BafA1 (10 nM) for 24 h. The experiment was repeated three times. **d**—Cell viability was recovered after 24 h exposure to Baf A1 (10 nM). The values are presented as the means ± SEM, **p < 0.05 vs. the control group, ^##^p < 0.05 vs. the FAC group (n = 6)

We examined the expression of LC3 as a marker of autophagy in iron-overloaded BMMNCs and found that the expression of LC3II increased with increasing FAC dose (Fig. 3a). Then, we examined iron-overloaded BMMNCs via confocal microscopy and found a significant increase in LC3-II expression and GFP-LC3-positive autophagosomes in the cells (Fig. 3b).

A basal level of autophagy has a protective effect on cells, but excessive autophagy can induce cell death. To clarify the effects of autophagy caused by iron overload on BMMNCs, we examined apoptosis. The results revealed no obvious apoptosis of iron-overloaded BMMNCs (data not shown). Next, iron-overloaded BMMNCs were treated with bafilomycin A1 (BafA1), an inhibitor of the proton pump of autophagic lysosomes. It can inhibit autophagy and reduce the degradation of autophagosomes. Under treatment with BafA1, the concentration of LC3II increased (Fig. 3c), suggesting that the increase in LC3II after iron overload promoted the increase in autophagic flow. Cell viability was also improved by treatment with BafA1 (Fig. 3d).

### Iron overload increases autophagy through mROS

**Fig. 4:**
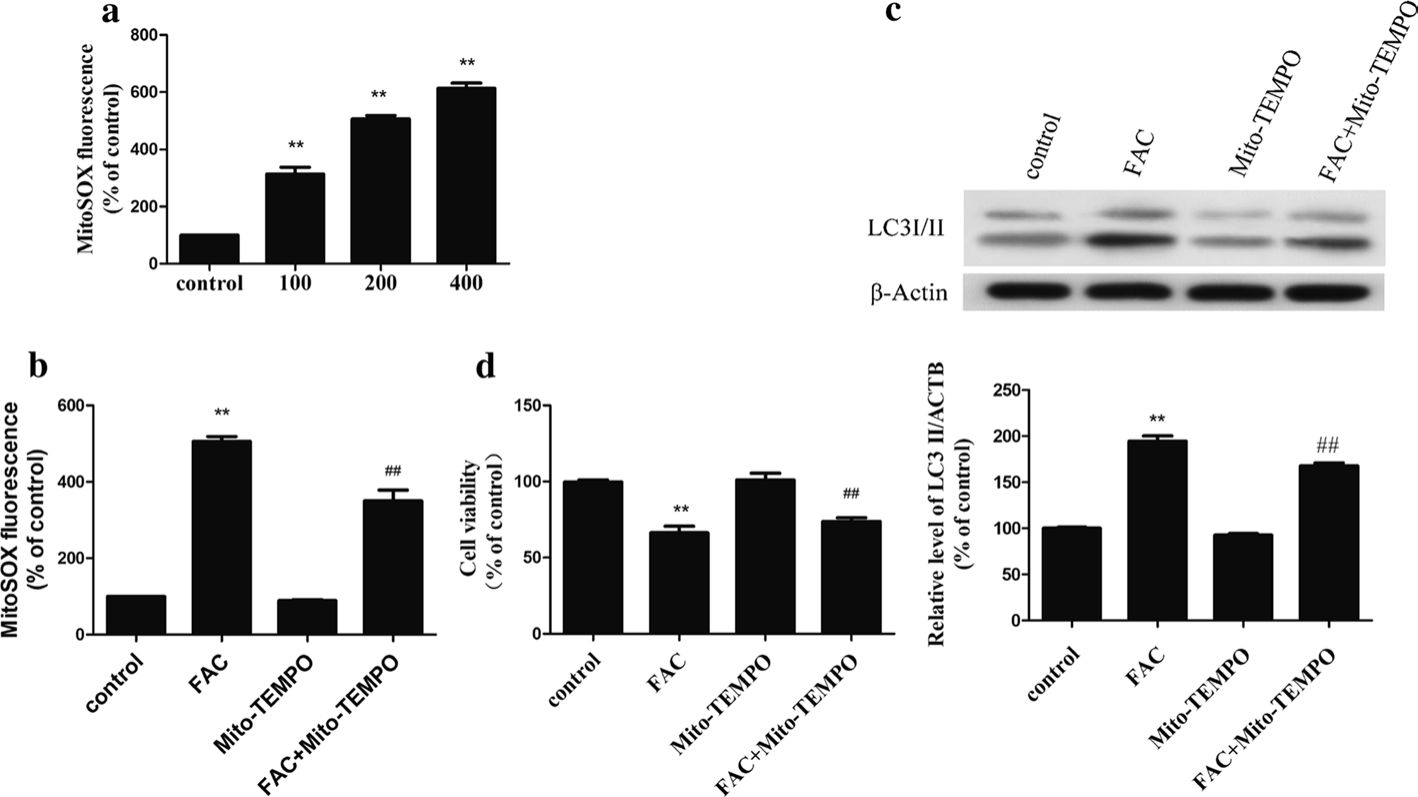
mROS mediates iron overload-induced autophagy in bone marrow mononuclear cells. **a**—Quantification of mROS levels using a fluorescence spectrometer after bone marrow mononuclear cells were treated with FAC at different concentrations for 24 h. **b** through **d**—Bone marrow mononuclear cells were preincubated with Mito-TEMPO (10 mM) for 2 h and then treated with 200 μM FAC, then the mROS levels (**b**), LC3 levels (**c**) and cell viability (**d**) were determined. The values are presented as the means ± SEM, **p < 0.05 vs. the control group, ^##^p < 0.05 vs. the FAC group

A relationship between autophagy and mROS has been reported [21]. The initial oxygen reduction product in mitochondria is O_2_, which is then rapidly converted to H_2_O_2_ [22]. Therefore, we examined the levels of mROS in iron-overloaded BMMNCs, and the results indicated significantly elevated mROS levels in these cells (Fig. 4a). In our animal experiment, we found that the mROS levels increased significantly in iron-overloaded group (Additional file 2: Fig. S3D). The levels of mROS were also found to be significantly higher in iron-overloaded patients than in healthy subjects (Fig. 2d).

BMMNCs were then pre-treated with the mitochondrial antioxidant Mito-TEMPO, which targets SOD in mitochondria, for 2 h before co-culture with FAC. Mito-TEMPO inhibited the production of mROS (Fig. 4b) and markedly inhibited the expression of LC3II caused by iron overload (Fig. 4c). Moreover, the viability of iron-overloaded cells was significantly improved by pre-treatment with Mito-TEMPO (Fig. 4d). TThese results suggest that mROS induces autophagy under iron-overloaded conditions, while antioxidants can reduce oxidative stress in mitochondria and thus reduce autophagy.

### Iron overload can increase SOD2 acetylation by inhibiting SIRT3 activity and expression

**Fig. 5:**
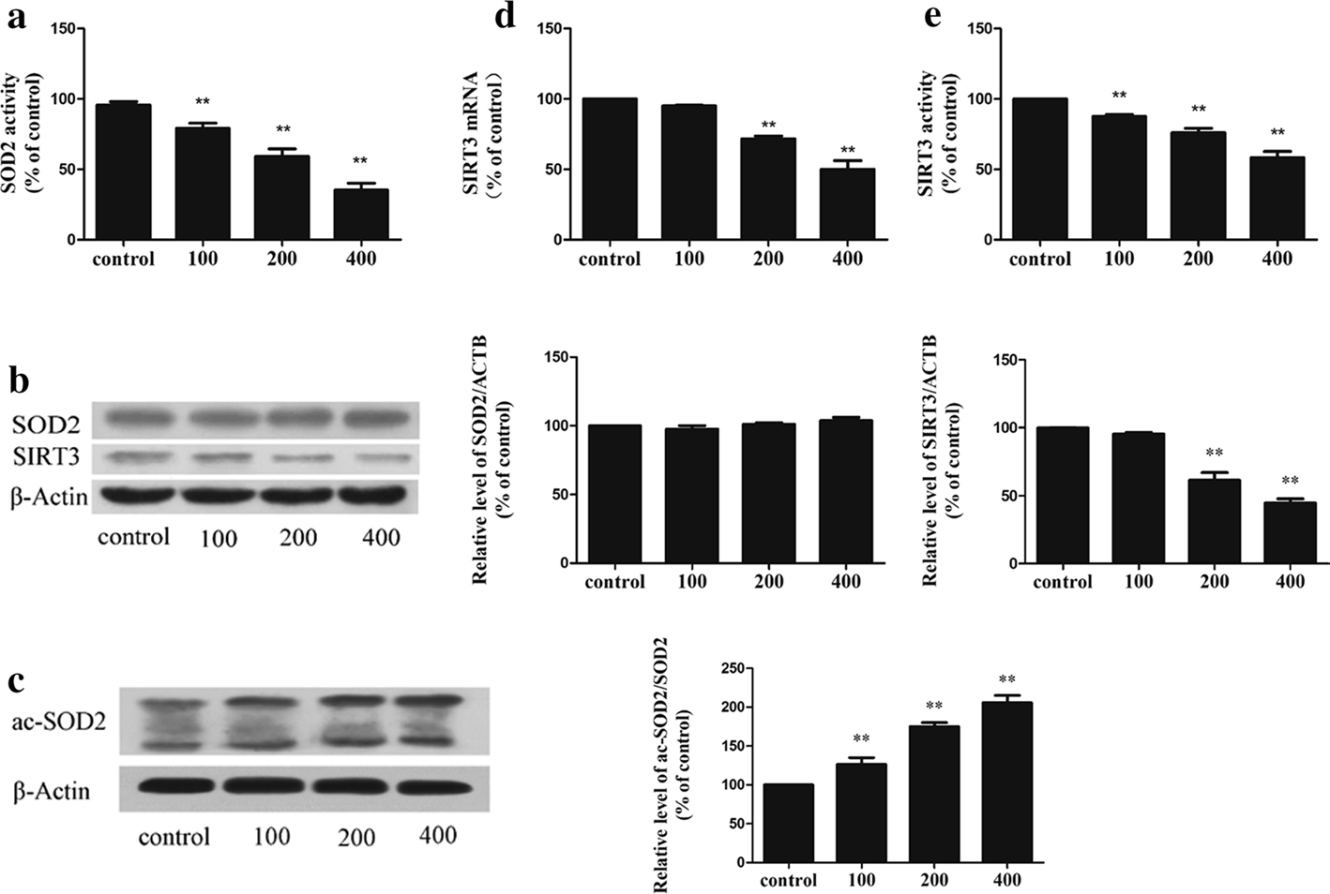
Iron overload can increase acetylated-SOD2 expression and decrease SIRT3 protein expression and activity in a dose-dependent manner. **a**—SOD2 activity in bone marrow mononuclear cells treated with FAC (μM). **b**—Representative immunoblot of SOD2 and SIRT3 protein levels in bone marrow mononuclear cells. β-actin was used as an internal standard for protein loading. **c**—Acetylation of SOD2 after FAC exposure was determined via immunoprecipitation with an anti-SOD2 antibody, followed by immunoblot analysis of acetylated-lysine. β-actin was used as an internal standard for protein loading. **d**—Quantitative real-time PCR analysis was applied to determine SIRT3 mRNA levels. **e**—SIRT3 activity was measured based on an enzymatic reaction using a SIRT3 assay kit. The values are presented as the means ± SEM, **p < 0.05 vs. the control group

SOD2 is the main scavenger of oxygen free radicals in the mitochondria. Therefore, we examined the effects of iron overload on SOD2 expression. Interestingly, the activity of SOD2 decreased with increasing FAC dose (Fig. 5a), but no effect was observed on the levels of SOD2 protein expression (Fig. 5b). SOD2 activity is regulated by the acetylation of lysine residues. We first detected the acetylation level of SOD2 by co-immunoprecipitation and western blotting. The results indicate that iron overload resulted in a progressive increase in the SOD2 acetylation level (Fig. 5c). The deacetylation of SOD2 is mainly regulated by the mitochondrial deacetylase, SIRT3 [5, 6, 23].

Therefore, we examined SIRT3 activity and concentration in iron-overloaded BMMNCs. Iron overloading resulted in significant decreases in the levels of SIRT3 mRNA (Fig. 5d) and protein (Fig. 5b). This study also showed that the levels of SIRT3 activity in the iron-overloaded group were significantly lower than in the control group (Fig. 5e).

We next performed iron overload mouse experiments and examined the bone marrow of iron-overloaded patients, and the results verified that SOD2 activity (Additional file 2: Fig. S3E) and SIRT3 (Additional file 2: Fig. S3F) activity were significantly lower under iron overload conditions. The activities of SOD2 (Fig. 2e) and SIRT3 (Fig. 2f) in BMMNCs from patients with iron overload were also significantly lower.

### SIRT3–SOD2 modulates iron overload-induced mROS accumulation and autophagy

**Fig. 6:**
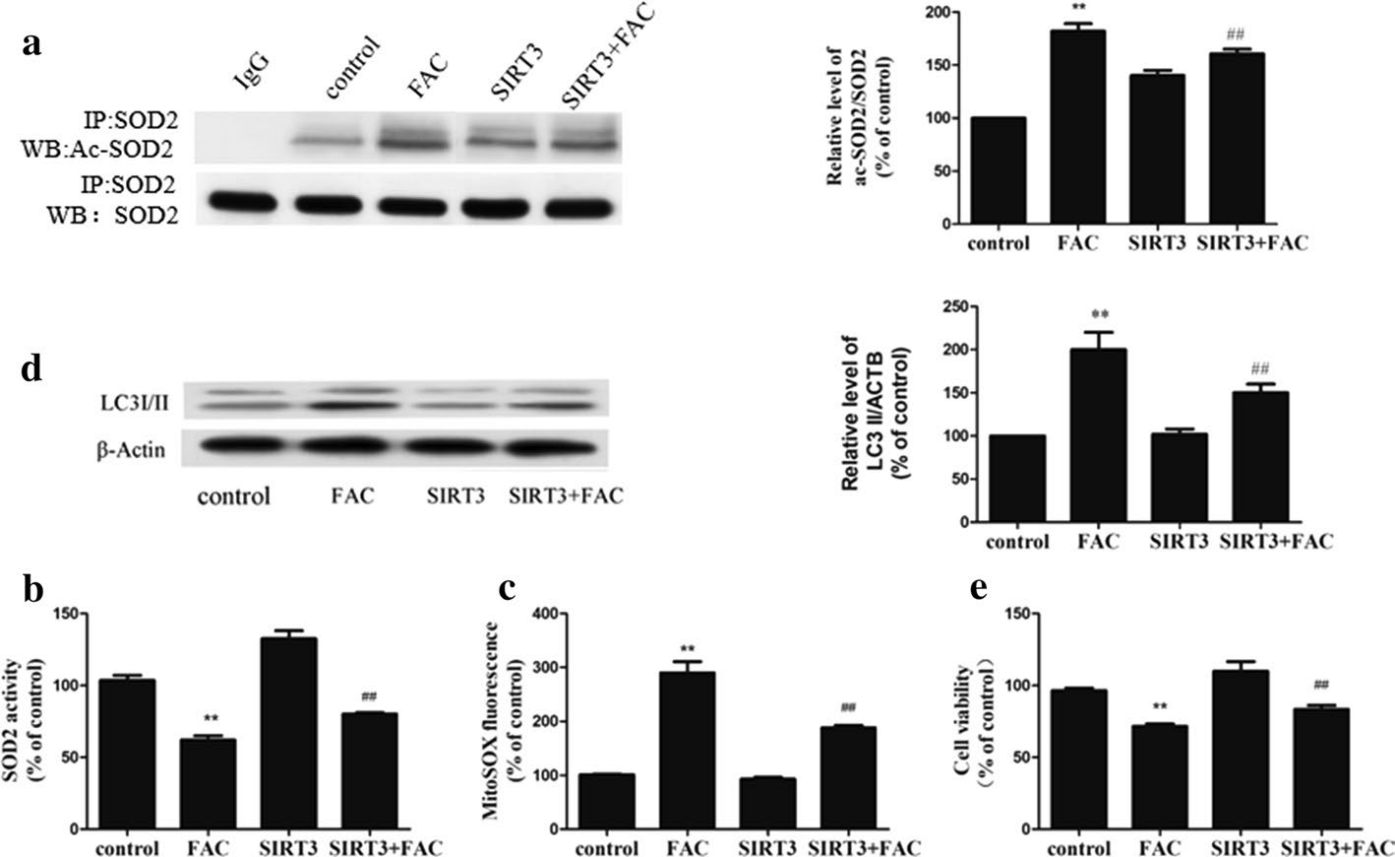
SIRT3–SOD2 modulates iron overload-induced mROS accumulation and autophagy in bone marrow mononuclear cells in vitro. **a**—SIRT3 overexpression induced ac-SOD2 after 200 μM FAC treatment. **b** through **e**—After SIRT3 overexpression, SOD2 activity in bone marrow mononuclear cells (**b**), mROS production in bone marrow mononuclear cells (**c**) and cell viability (**e**) were tested. **d**—A representative immunoblot of LC3 protein levels in bone marrow mononuclear cells. β-actin was used as an internal standard for protein loading. The values are presented as the means ± SEM, **p < 0.05 vs. the control group, ^##^p < 0.05 vs. the FAC group

Transfection-induced overexpression of SIRT3 in BMMNCs ameliorated the inhibition of SIRT3 activity induced by iron overload (data not shown), reduced the acetylation of SOD2 (Fig. 6a) and increased the activity of SOD2 (Fig. 6b), effectively reducing the mROS produced by iron overload (Fig. 6c). SIRT3 overexpression also significantly reduced LC3II expression in the overloaded cells and increased their viability (Fig. 6d, e, respectively).

### Curcumin protects against iron overload-induced autophagic cell death

**Fig. 7:**
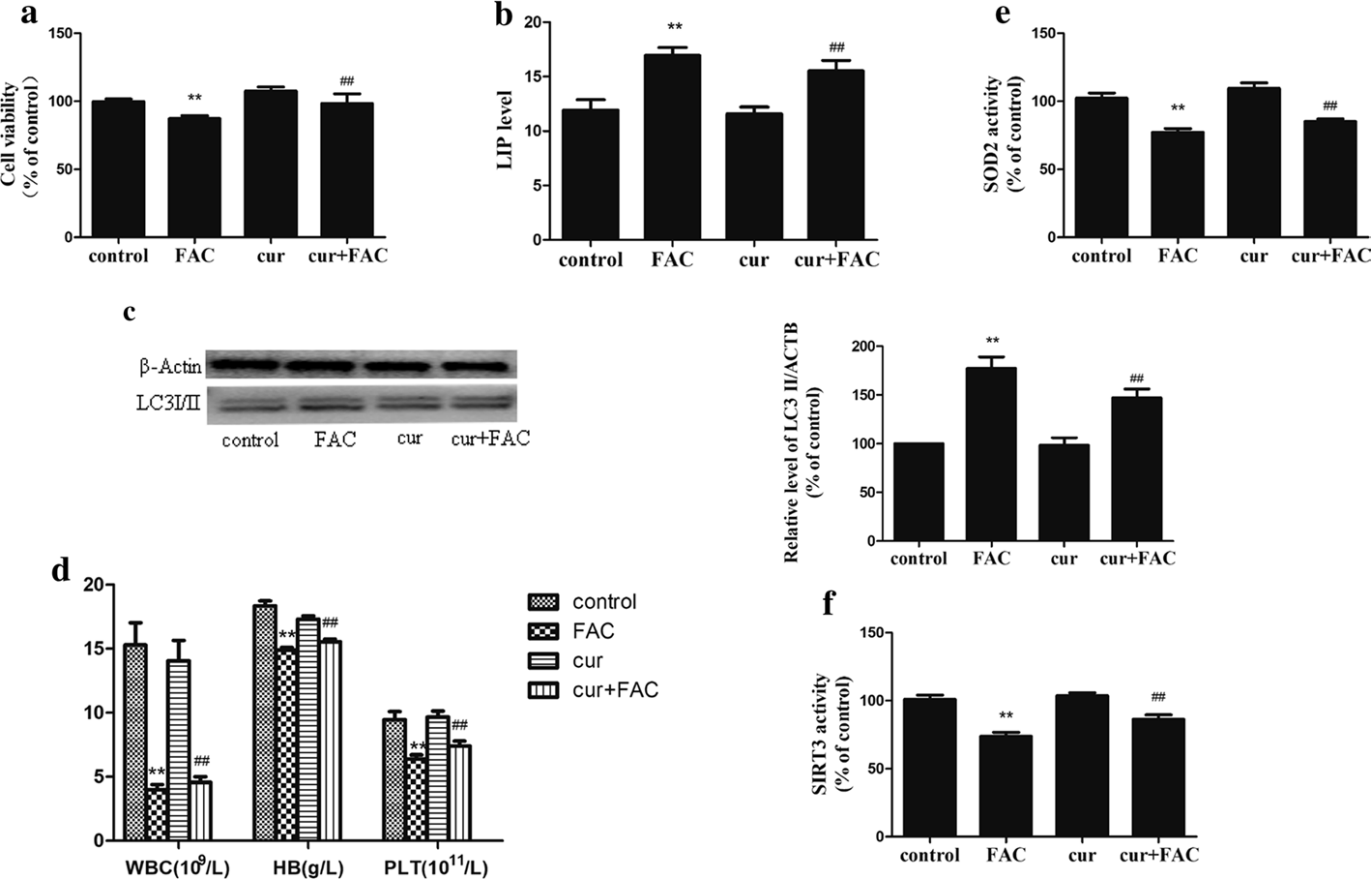
Curcumin protects against iron overload-induced autophagic cell death in vivo (n = 15). **a**—Cell viability. **b**—LIP in the cells. **c**—A representative immunoblot analysis of LC3. **d**—Peripheral blood test. **e**, **f**—SOD2 (**e**) and SIRT3 (**f**) activity in mice treated with curcumin. The results are expressed as a percentage of the control group, which is set at 100%. The values are presented as the means ± SEM, **p < 0.05 vs. the control group, ^##^p < 0.05 vs. the FAC group

The antioxidant curcumin [24] reduced the elevation of mROS induced by iron exposure (Additional file 3: Fig. S2A), while there was no significant decrease in intracellular LIPs (Additional file 3: Fig. S2B). Curcumin partially recovered cell viability (Additional file 3: Fig. S2C) and reduced the expression of LC3II (Additional file 3: Fig. S2D). In the mouse model, cell viability was markedly better in the iron-overloaded + curcumin group than in the iron-overloaded group (Fig. 7a). Furthermore, the LIPs in the cells were similar (Fig. 7b), the LC3II level significantly decreased (Fig. 7c), and the haemoglobin, platelet count and white blood cell count were elevated (Fig. 7d; Table 1).

**Table 1:** Peripheral blood cell indices of mice

Group	WBC (× 10^9^/l)	HB (g/l)	PLT (× 10^11^/l)
Control	15.2 ± 5.2	18.4 ± 1.3	9.3 ± 2.1
Cur	14.1 ± 4.8	17.3 ± 0.8	9.7 ± 1.5
FAC	4.0 ± 1.2*	14.9 ± 0.7*	6.3 ± 1.0*
Cur + FAC	4.6 ± 1.4^#^	15.6 ± 0.6^#^	7.4 ± 1.2^#^

*WBC* white blood cell, *HB* hemoglobin, *PLT* platelet, *Cur* curcumin group, *FAC* iron-overloaded group, *Cur + FAC* curcumin + iron-overloaded group

^*^p < 0.05 versus the control group

^#^p < 0.05 vs. the FAC group. (n = 15)

Iron-overloaded group (200 mg/kg iron dextrin intraperitoneal injection, 1 ml/kg corn oil gavage, n = 15); control group (0.2 ml normal saline intraperitoneal injection, 1 ml/kg corn oil gavage, n = 15); curcumin group (0.2 ml normal saline intraperitoneal injection, 200 mg/kg curcumin dissolved in corn oil gavage, n = 15); and curcumin + iron-overloaded group (200 mg/kg iron dextrin intraperitoneal injection, 200 mg/kg curcumin dissolved in corn oil gavage, n = 15).

### Curcumin inhibits iron overload-induced autophagy through a SIRT3–SOD2-dependent mechanism

**Fig. 8:**
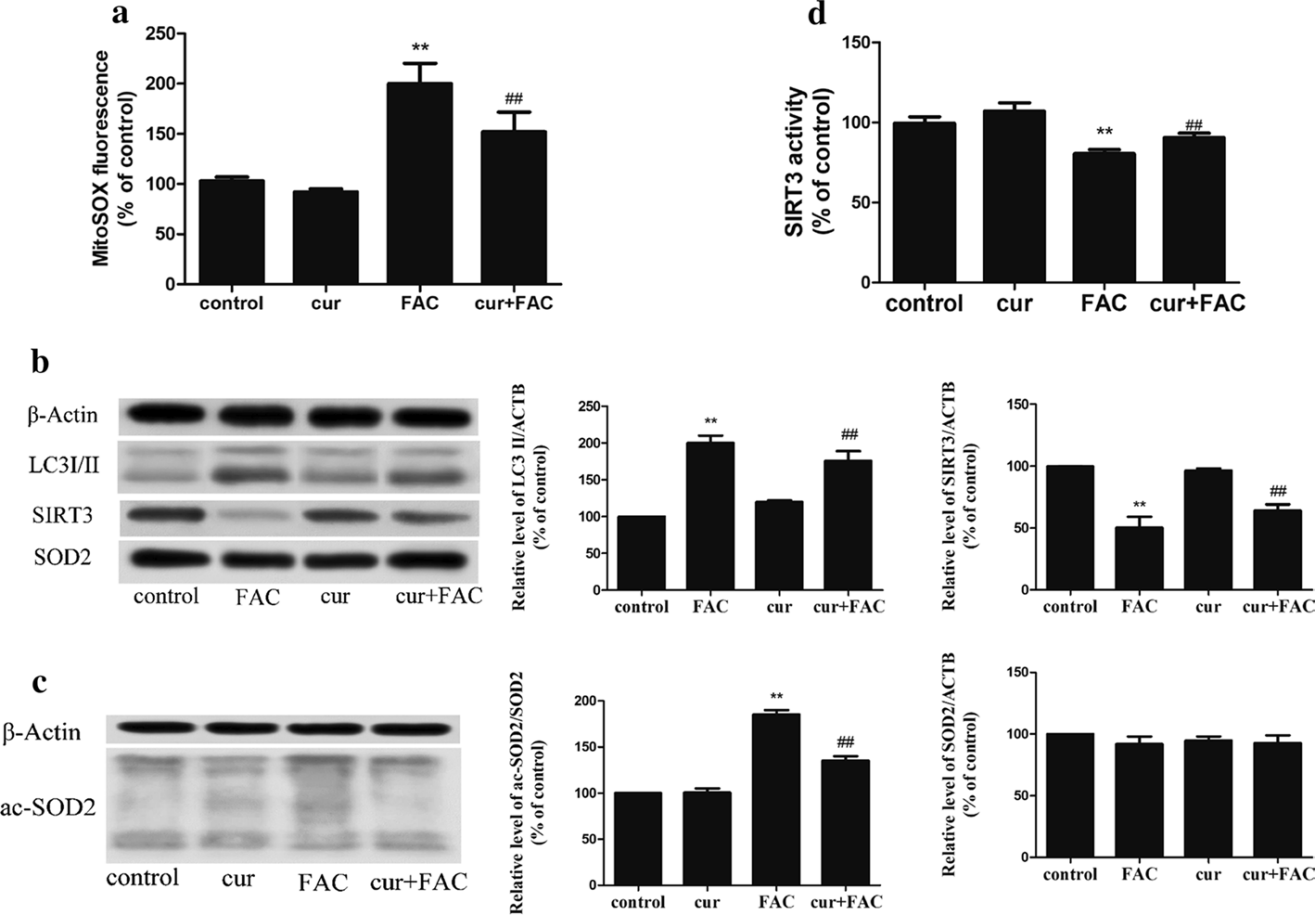
Curcumin pre-treatment reduces iron overload-induced autophagy in bone marrow mononuclear cells in vitro. **a**—The effects of curcumin pre-treatment on mROS production. **b**, **c**—A representative immunoblot analysis of SIRT3 and SOD2 protein (**b**) and SOD2 acetylation (**c**). **d**—The effects of curcumin pre-treatment on SIRT3 activity. The values are presented as the means ± SEM, **p < 0.05 vs. the control group, ^##^p < 0.05 vs. the FAC group

SIRT3 is related to the autophagy and bone marrow damage caused by iron overload. Interestingly, curcumin partially restored the inhibition of SIRT3 activity induced by iron overloading (Fig. 8d) and the SIRT3 protein level (Fig. 8b). As expected, curcumin pre-treatment reduced mROS production (Fig. 8a), inhibited the SOD2 acetylation (Fig. 8c) induced by iron overloading, and restored SOD2 activity, but it had a minimal effect on the level of SOD2 protein (Fig. 8b). In the mouse model, the SIRT3 activity for the iron overload + curcumin group was significantly higher than that of the iron overload group (Fig. 7f), and SOD2 activity was restored (Fig. 7e).

## Discussion

Iron overload is a common complication in haematological patients. Numerous studies have shown that iron overload has negative effects on haematogenesis [25, 26]. Iron chelation was shown to decrease transfusion requirements and increase platelet and neutrophil counts, thus providing indirect evidence for the toxic effect of iron overload on haematogenesis [25–27]. Furthermore, haematopoietic progenitor cells also showed suppressed colony-forming capacity under conditions of iron overload [27, 28]. Our study indicates that iron-overloaded BMMNCs have decreased viability and cell proliferation activity both in vitro and in vivo, but no obvious apoptosis was observed. The haemoglobin, platelet count and white blood cell count in peripheral blood were markedly lower in iron-overloaded mice than in control mice.

Many studies have indicated that iron overload leads to increased ROS levels. Haematopoietic stem cells are predominantly present in the low oxygen milieu of the bone marrow, and high levels of ROS are harmful to normal haematogenesis [29, 30]. However, ROS are also known to induce autophagy. Appropriate levels of autophagy are necessary to maintain homeostasis, but excessive levels of autophagy promote cell death [14]. Few reports have addressed whether autophagy occurs in iron overload.

Here, we show that iron overload disrupts normal haematogenesis and leads to increased mROS levels. These observations were consistent with the results of other in vitro studies [31–33].

Although mitochondrial oxidative stress is associated with autophagy, the molecular mechanism underlying the accumulation of oxygen free radicals from the mitochondria remains unclear. Cells rigorously regulate the oxygen free radicals derived from mitochondria. Mitochondria have specific mechanisms for removing excess oxygen free radicals to maintain homeostasis. SOD2 is only present in the mitochondria and is involved in scavenging free radicals [34, 35]. As mitochondria consume 90% of intracellular oxygen, SOD2 activity is very important to maintain the balance of oxygen free radicals derived from mitochondria [36, 37]. The amount of SOD2 protein is regulated at the level of transcription, and its activity is regulated by acetylation [36–38]. Consistent with previous studies, here, iron overload resulted in an elevated level of SOD2 acetylation in a dose-dependent manner. These data suggest that iron overload increases mitochondrial oxygen free radicals by increasing SOD2 acetylation levels, rather than by decreasing SOD2 protein expression.

Lysine acetylation is an important post-translational event in the regulation of mitochondrial proteins and autophagy [39–41]. SIRT3 is the strongest deacetylase in the mitochondria. It is directly involved in mitochondrial energy synthesis and in the control of oxygen free radical levels [42]. SIRT3 regulates SOD2 activity by regulating the acetylation level of SOD2 and its target lysine has been identified. SIRT3 deacetylates SOD2 in response to ionizing radiation, indicating that SOD2 is a major downstream signal of SIRT3-mediated mitochondrion-derived oxygen reduction [38]. Our findings indicate that iron overload did not lead to a decrease in SOD2 expression, but instead to an increase in acetylation levels. We also found decreases in both SIRT3 expression and activity. SIRT3 overexpression was shown to ameliorate the increase in SOD2 acetylation caused by iron overload. These findings indicate that SIRT3–SOD2-mediated autophagy is an important mechanism in the bone marrow injury induced by iron overload.

Recent studies [43, 44] have shown that curcumin, a natural antioxidant extracted from the rhizome of Zingiberaceae plants, can significantly alleviate oxidative stress damage through mitochondrial subunit localization. Waseem et al. reported that curcumin significantly alleviated cisplatin-induced oxidative damage in liver and brain tissues via the mitochondrial pathway [45]. Our results show that curcumin can restore the activity of SIRT3, reduce the level of SOD2 acetylation, and restore the activity of SOD2, thereby reducing the production of mROS.

## Conclusion

We propose a possible mechanism whereby iron overload induces bone marrow damage via mROS-dependent autophagy. Curcumin shows a protective role in eliminating mROS and suppressing autophagy through the SIRT3–SOD2 pathway, both in vitro and in vivo. These findings provide new insight into the link between curcumin and autophagy signalling, which could contribute to a better understanding of the protective effect in iron overload-induced bone marrow damage.

## Supplementary Information

**Additional file 1: Fig. S1.** Iron overload could cause bone marrow damage in vitro. A through C—FAC (μM) exposure decreases the cell viability (A) and cell proliferation activity (B) of bone marrow mononuclear cells in a dose-dependent manner. The intracellular LIPs increased accordingly (C). The values are presented as the means ± SEM, **p < 0.05 vs. the control group.

**Additional file 2: Fig. S3.** Iron overload could cause bone marrow damage in vivo. A—The cell viability of mononuclear cells in the bone marrow of the iron-overloaded mice decreased. B and C—LIP (B) and mROS (C) levels increased in iron-overloaded mice. D and E—SOD2 (D) and SIRT3 (E) activities decreased in iron-overloaded mice. F—Iron-overloaded mice showed decreased Hb levels, PLT counts, and WBC counts in their peripheral blood. The values are presented as the means ± SEM, **p < 0.05 vs. the control group.

**Additional file 3: Fig. S2.** Curcumin suppresses iron overload-induced autophagic cell death in vitro. A—Curcumin reduced the elevation of mROS induced by iron overload. B—Intracellular LIPs did not decrease significantly. C—Curcumin partially recovered cell activity. D–A representative immunoblot analysis of LC3. The values are presented as the means ± SEM, **p < 0.01 vs. the control group, ^##^p < 0.01 vs. the FAC group (n = 6).

## Abbreviations

mROS: Mitochondrial reactive oxygen species
LIP: Labile iron pools
LC3: Microtubule-associated protein 1 light chain 3
ROS: Reactive oxygen species
SIRT3: Sirtuin 3
SOD2: Superoxide dismutase 2
BMMNC: Bone marrow mononuclear cell
EDTA: Ethylenediaminetetraacetic acid
WBCs: White blood cells
Hb: Haemoglobin
RBCs: Red blood cells
PLTs: Platelets
FBS: Foetal bovine serum
BSA: Bovine serum albumin
FAC: Ferric ammonium citrate
CFU-E: Colony-forming unit erythroid
BFU-E: Burst-forming unit erythroid
CFU-GM: Colony-forming unit granulocyte–macrophages
CFU-Mix: Colony-forming unit Mix
BIP: Bipyridine
BafA1: Bafilomycin A1
