# Supplementary figures and images for: Iron overload adversely effects bone marrow haematogenesis via SIRT-SOD2-mROS in a process ameliorated by curcumin
Source: Cell Mol Biol Lett. 2021 Jan 13;26:2. doi: 10.1186/s11658-020-00244-7 (PMC7805071; doi:10.1186/s11658-020-00244-7)

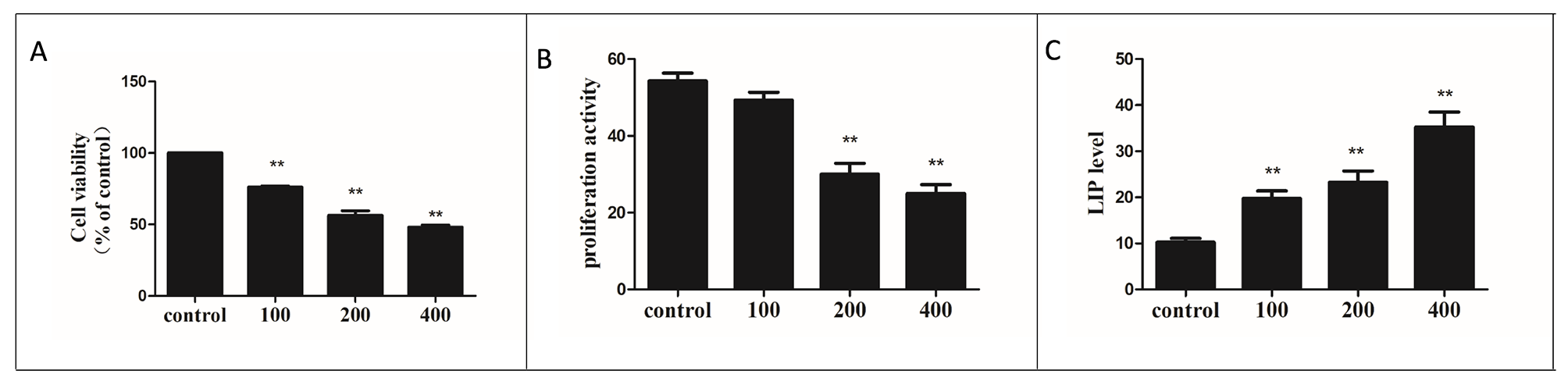

Supplement: Supplementary file 1 — Additional file 1: Fig. S1. Iron overload could cause bone marrow damage in vitro. A through C—FAC (μM) exposure decreases the cell viability (A) and cell proliferation activity (B) of bone marrow mononuclear cells in a dose-dependent manner. The intracellular LIPs increased accordingly (C). The values are presented as the means ± SEM, **p < 0.05 vs. the control group. [file 11658_2020_244_MOESM1_ESM.tif]

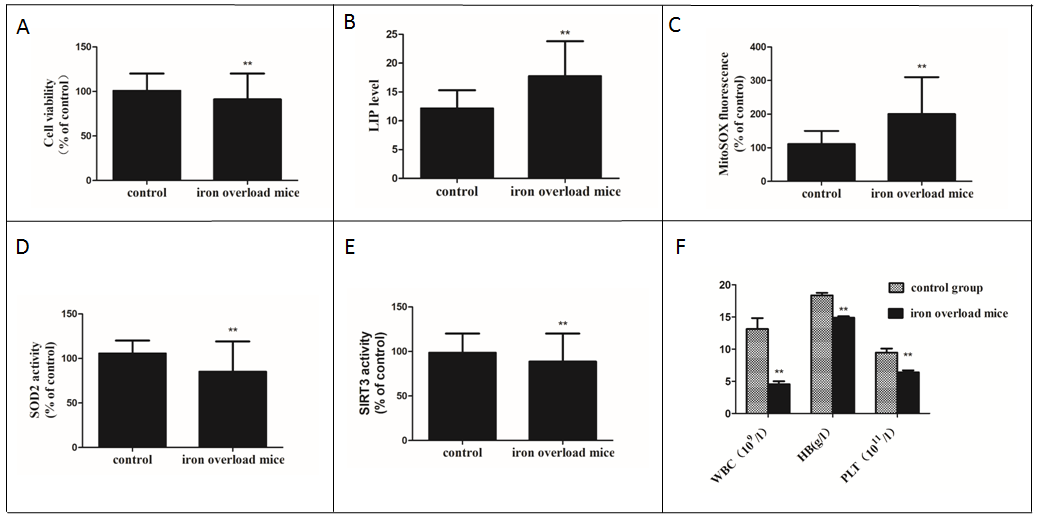

Supplement: Supplementary file 2 — Additional file 2: Fig. S3. Iron overload could cause bone marrow damage in vivo. A—The cell viability of mononuclear cells in the bone marrow of the iron-overloaded mice decreased. B and C—LIP (B) and mROS (C) levels increased in iron-overloaded mice. D and E—SOD2 (D) and SIRT3 (E) activities decreased in iron-overloaded mice. F—Iron-overloaded mice showed decreased Hb levels, PLT counts, and WBC counts in their peripheral blood. The values are presented as the means ± SEM, **p < 0.05 vs. the control group. [file 11658_2020_244_MOESM2_ESM.tif]

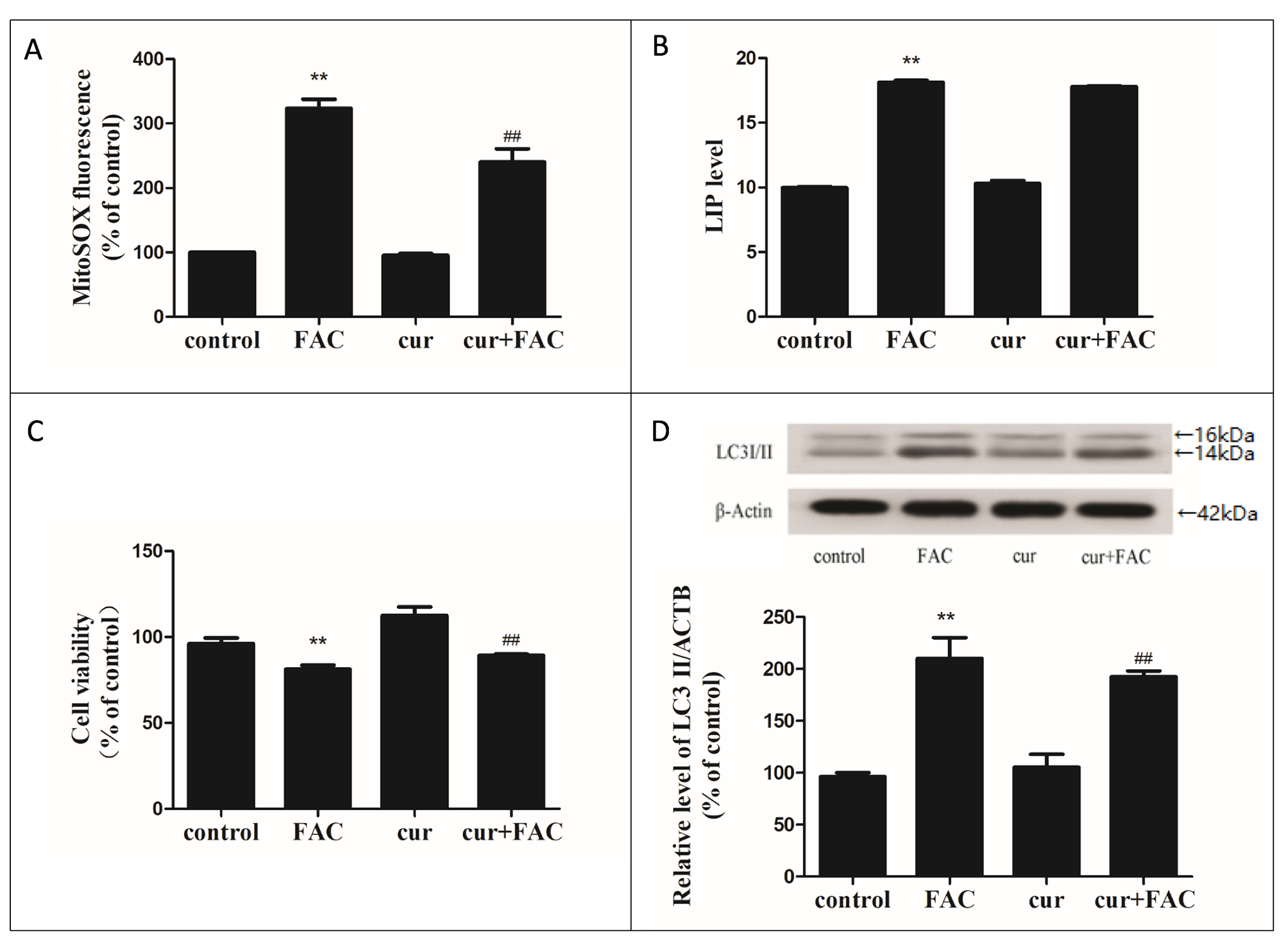

Supplement: Supplementary file 3 — Additional file 3: Fig. S2. Curcumin suppresses iron overload-induced autophagic cell death in vitro. A—Curcumin reduced the elevation of mROS induced by iron overload. B—Intracellular LIPs did not decrease significantly. C—Curcumin partially recovered cell activity. D–A representative immunoblot analysis of LC3. The values are presented as the means ± SEM, **p < 0.01 vs. the control group, ##p < 0.01 vs. the FAC group (n = 6). [file 11658_2020_244_MOESM3_ESM.tif]
